# Diffusion of solutes inside bacterial colonies immobilized in model cheese depends on their physicochemical properties: a time-lapse microscopy study

**DOI:** 10.3389/fmicb.2015.00366

**Published:** 2015-04-30

**Authors:** Juliane Floury, Ilham El Mourdi, Juliana V. C. Silva, Sylvie Lortal, Anne Thierry, Sophie Jeanson

**Affiliations:** ^1^INRA, UMR1253 Science and Technology of Milk and EggsRennes, France; ^2^Agrocampus Ouest, UMR1253 Science and Technology of Milk and EggsRennes, France

**Keywords:** bacterial colony, *Lactoccocus lactis*, cheese, confocal microscopy, diffusion, milk protein

## Abstract

During cheese processing and ripening, bacteria develop as colonies. Substrates and metabolites must then diffuse either from or into the colonies. Exploring how the inner cells of the colony access the substrates or get rid of the products leads to study the diffusion of solutes inside bacterial colonies immobilized in cheese. Diffusion limitations of substrates within the bacterial colony could lead to starvation for the cells in the center of the colony. This study aimed at better understands ripening at the colony level, by investigating how diffusion phenomena inside colonies vary depending on both the physicochemical properties of the solutes and *Lactococcus lactis* strain. Dextrans (4, 70, and 155 kDa) and milk proteins (BSA, lactoferrin and α_S1_-casein) of different sizes and physicochemical properties were chosen as model of diffusing solutes, and two *L. lactis* strains presenting different surface properties were immobilized as colonies in a model cheese. Diffusion of solutes inside and around colonies was experimentally followed by time-lapse confocal microscopy. Dextran solutes diffused inside both lactococci colonies with a non-significantly different effective diffusion coefficient, which depended mainly on size of the solute. However, whereas flexible and neutral hydrophilic polymers such as dextran can diffuse inside colonies whatever its size, none of the three proteins investigated in this study could penetrate inside lactococci colonies. Therefore, the diffusion behavior of macromolecules through bacterial colonies immobilized in a model cheese did not only depends on the size of the diffusing solutes, but also and mainly on their physicochemical properties. Milk caseins are probably first hydrolyzed by the cell wall proteases of *L. lactis* and/or other proteases present in the cheese, and then the generated peptides diffuse inside colonies to be further metabolized into smaller peptides and amino acids by all the cells located inside the colonies.

## Introduction

During cheese making, regardless of the cheese type, bacteria are immobilized in the curd during the coagulation step, and then grow as colonies spread within the cheese curd. Jeanson et al. (2011) showed that the distribution of *Lactococcus lactis* colonies was random in a non-fat model cheese. Lactococci are the most used starters in the cheese industry. They are a major actor of ripening which gives the cheese its final sensorial properties. During ripening, they are responsible for the proteolysis, the milk protein breakdown, leading to peptides and amino acids. It is then obvious that the access to nitrogen sources, i.e., proteins and derivates, is of major importance for the proteolysis activity and the bacterial metabolism of the cells within the colony. However, the way the bacteria interact with dairy components is still poorly understood (Burgain et al., 2014). It is highly probable that, on one hand, milk proteins have to diffuse from the cheese matrix (a fat-protein network) into the colony to reach the bacterial cells in the center of the colony. Indeed, nutrients have to reach the center cells of the colony; otherwise these center cells may be starved. On the other hand, proteolysis end-products (small peptides and amino acids) have to diffuse from the bacterial colonies into the cheese matrix. If diffusion limitations occur inside the bacterial colony, gradients of concentration of both nitrogen sources (low concentrations in the center of the colony) and nitrogen end-products (high concentrations in the center of the colony) may be generated and may affect the metabolic activity of microbial cells, and thus the kinetics of the ripening process. The mean diameters of colonies and the mean distance between them in a model cheese were shown to be strongly influenced by the initial inoculation level. The lower the inoculation level was, the larger the colonies were, and then the further away they were from each other (Jeanson et al., 2011). It has previously been observed that low concentration of substrates could generate different physiological states or different growth rates in pathogenic bacterial colonies when colonies were bigger than 400 μm diameter (McKay et al., 1997; Kreft et al., 1998). The main hypothesis for these observations was that the diffusion limitations of substrates within the bacterial colony lead to starvation for the cells in the center of the colony. As a consequence, lysis could be higher for the center cells in *Vibrio cholera* colonies (Wimpenny, 1992). If lysis occurs at the center of the colony, it is also very important to know if bacterial enzymes could diffuse out of the colony to determine how far from the colony proteolytic enzymes could diffuse outside the colony, in the cheese matrix.

However, Floury et al. (2010) reported a strong lack of data about the diffusion properties of key molecules like sugars, organic acids, proteins, and peptides in cheese. The first effective diffusion coefficient was determined for nisin in model cheeses (Aly et al., 2011). Using fluorescently labeled solutes, Silva et al. (2013) showed that dextran macromolecules up to 2 MDa, and different dairy proteins, were able to diffuse through the model cheese. Quite interestingly, the proteins tested (rigid and negatively charged molecules) were hindered to a greater degree than the dextrans (flexible and neutral molecules) in the model cheese, due to specific interactions between the protein matrix and the diffusing proteins. So far, only our previous study (Floury et al., 2013) has investigated the diffusion of molecules within bacterial colonies. Even if it has been demonstrated that viral particles such as bacteriophages (≈100 nm head) could diffuse inside biofilms (Lacroix-Gueu et al., 2005; Briandet et al., 2008), we consider that the structure of biofilms (with exopolysaccharide matrix) and colonies is not really comparable. It was then very important to understand ripening at the colony level by investigating the diffusion of model nutrient macromolecules, such as polysaccharides and milk proteins, inside lactococci colonies.

In our previous study (Floury et al., 2013), we developed a specific experimental design and we demonstrated for the first time that model solutes of different sizes (dextran macromolecules from 4.4 to 155 kDa) were able to diffuse inside bacterial colonies of *L. lactis*, immobilized in two different models of solid food matrices (model cheese and agar). The principle of this static design was to deposit a solution of the fluorescently-labeled diffusing solute on the upper side of a gel cassette (Brocklehurst, 1995) filled with the solid medium, previously inoculated with *L. lactis*. The gel cassettes were then directly observed by confocal laser microscopy and the corresponding relative fluorescence intensity profiles within the colony vs. in the surrounding media were quantified after 3 h of diffusion of the fluorescent solutes at 19°C. It was concluded that colonies of *L. lactis* LD61 immobilized in the model cheese were porous to all dextrans from 4 to 155 kDa after this delay of migration, but no kinetic aspect of diffusion could be assessed.

The objective of the present work was to determine how diffusion phenomena inside colonies vary depending on both the properties of solutes and *L. lactis* strain. We quantified the diffusion rates of solutes of different sizes and physico-chemical properties both around and inside colonies immobilized in a model cheese, for two *Lactococcus lactis* strains presenting different surface properties. Our experimental device was improved by adapting the time-lapse microscopy method described in Rani et al. (2005), originally developed to determine the effective diffusion coefficients of fluorescent tracers into biofilm cell clusters of *Staphylococcus epidermis* and their surrounding solution.

## Materials and methods

### Bacterial strains and growth conditions

*Lactococcus lactis* subsp. *lactis* biovar *diacetylactis* LD61 was used (collection of the Centre International de Resources Microbiennes–Bactéries d'Intérêt Alimentaire (CIRM-BIA), INRA, Rennes, France) and was routinely grown under static conditions in M17 lactose broth (Difco, Becton Dickinson, Le Pont de Claix, France) at 30°C.

*Lactococcus lactis* subsp. *lactis* TIL1230 was kindly given M-P Chapot-Chartier and obtained from the parental strain NCDO2110 (Giaouris et al., 2009). This strain was lactose and protease negative and was then grown under static conditions in M17 lactose broth supplemented with 0.5% glucose (Sigma) at 30°C. Therefore, to ensure its optimal growth in milk, the milk cultures of TIL1230 were supplemented with 1% glucose (Sigma) and 0.3% peptone casein (BD).

### Bacterial surface characterization

#### Cell surface hydrophobicity

Net surface charge of the bacteria and the presence of lipophilic compounds affect partitioning between two immiscible liquids (Burgain et al., 2014). The microbial adhesion to solvents (MATS) method was employed for the evaluation of the hydrophobic/hydrophilic character of the cell surface of *L. lactis* strains and for their Lewis acid–base characteristics. On this basis, we selected chloroform, a monopolar and acidic solvent (electron acceptor) and hexadecane, an apolar alkane using the protocol fully described in Giaouris et al. (2009). The values of MATS obtained with the chloroform were regarded as a measure of electron donor/basic characteristics of bacteria. Adhesion ability of the bacteria to the solvent is expressed as a percentage (%) according to the following relation:

(1) $$\%\;adhesion\;=\;\frac{OD_{400}^{initial\;aqueous\;phase}-OD_{400}^{aqueous\;phase\;after\;mixing}}{OD_{400}^{initial\;aqueous\;phase}}$$

With OD the optical density of the bacterial suspension measured at 400 nm. Each measurement was performed in triplicate and the experiment was repeated twice with independent bacterial cultures.

#### Cell surface charge

The electrophoretic mobility (EM) was measured to determine the cell surface net charge of the two bacteria according to the protocol described in Boonaert and Rouxhet (2000). EM of the bacteria with the appropriate pH values were measured at room temperature on a Zetameter model (Zeta Sizer Nano Series, Malvern Instruments Ltd, Malvern, UK). Experiments were made twice with independent culture with triplicate measurements. EM was expressed in 10^−8^ m^2^/V.s.

### Preparation of the model cheese in imaging chambers

A fat-free cheese made from renneted concentrated skim milk was used as model cheese, as previously described in Floury et al. (2013). This non-fat model cheese has the great advantage over traditional cheese technology to be a repeatable and homogeneous cheese matrix. Moulded after renneting, it is coagulated without further syneresis of the gel, thus exhibiting highly reproducible micro- and macro- structural properties. The concentrated milk was inoculated for a final concentration of 10^5^ CFU/ml, and coagulant agent (Maxiren 180; DSM Food Specialities, Seclin, France) was added at a final concentration of 300 μl/l. After homogenization, 400 μl of the mixture was slowly poured into several CoverWell imaging chambers (Sigma-Aldrich, Saint-Quentin Fallavier, France) that allow direct observation under the confocal microscope (Floury et al., 2012). The imaging chambers with the model cheese were then vertically incubated at 30°C for 15 h for coagulation and growth of the *L. lactis* LD61. For the *L. lactis* TIL1230, imaging chambers were also vertically incubated, for 8 h at 30°C and then for 15 h at 19°C. In parallel, the same media were also inoculated in 30 ml-bottles to measure the pH during acidification by *L. lactis*. The pH of the model cheeses were 5.05 ± 0.06, and 5.32 ± 0.10 for LD61 and TIL1230, respectively, after 15 h of incubation.

### Fluorescent dyes and labeled solutes

SYTO9™ was added before coagulation of the model cheese to a final concentration of 1.2 μmol/l, in order to dye the bacterial cells and to visualize colonies within the opaque matrix of cheese. SYTO9™ penetrates all bacterial membranes and dies all the bacterial cells, alive, and damaged (Boulos et al., 1999).

Three Rhodamine B isothiocyanate (RITC) conjugated dextrans of 10, 70, and 155 kDa were chosen as model of flexible and neutral polymers of anhydroglucose of different sizes (Table 1), labeled with an extent of labeling from 0.002 to 0.015 mol RITC per mol glucose (Sigma-Aldrich, Saint-Quentin Falavier, France). RITC-dextrans were dissolved to 50 mg/ml in distilled water.

**Table 1:** **Physicochemical properties of the fluorescently-labeled solutes**.

	**Dextran**			**BSA**	**Lactoferrin**	**α_S1_-casein**
	**10**	**70**	**155**			
Molecular weight (kDa)	10	70	155	66.4^a^	77^b^	23.6^c^
Isolectrical point	-	-	-	≈5^a^	8–9^b^	4.94^d^
Hydrodynamic radius (nm)	2.3^e^	6^e^	8.5^e^	3.65	2.2^f^	2.9^c^
Flexibility	flexible			rigid		flexible
Hydrophobicity	hydrophilic			hydrophobic		amphiphile

a Böhme and Scheler (2007);

b Bokkhim et al., (2013);

c Marchin et al. (2007);

d –values reported by expasy.org;

e –values reported by Sigma-Aldrich; supplier data online;

f *Chaufer et al. (2000)*.

The studied set of solutes was completed with three milk proteins, one random coil milk protein, the α_S1_-casein (INRA, Rennes, France), and two globular dairy proteins, bovine serum albumin (BSA, Sigma-Aldrich, Saint-Quentin Falavier, France) and lactoferrin (LF, Fonterra Boulogne-Billancourt, France). The proteins were labeled with free RITC (Sigma) using the protocol described in Silva et al. (2013). The three solutions were lyophilized and labeling efficiencies were determined by mass spectroscopy. BSA, LF, and α_S1_-casein were mainly mono-labeled with RITC. Finally, the RITC-labeled solutes were either dissolved to 50 mg/ml in water for the dextrans and the lactoferrin, in a permeate solution obtained from the ultrafiltration of skimmed milk for α_S1_-casein, and in a 0.1 M BisTris buffer at pH 6.8 for the BSA.

The solutions of labeled solutes were stored at −20°C, protected from light before and during fluorescence measurements.

Physicochemical properties of the solutes are summarized in Table 1.

### Experimental device for solute diffusion

#### Experimental set-up

**Figure 1:**
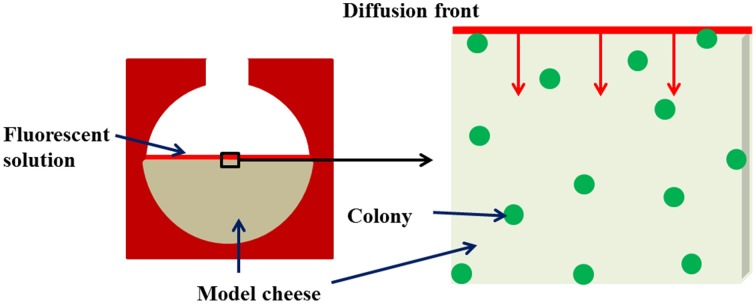
**Diagram of the experimental device for monitoring the kinetics of diffusion of solutes around and inside colonies immobilized in the model cheese**.

After the incubation time necessary for bacterial growth, a concentration gradient of the fluorescently-labeled solutes between the surface of the imaging chamber and the model cheese was triggered in order to induce the diffusion phenomenon. Five microliter of the fluorescently-labeled dextran or protein solution was dropped off at the surface of the coagulated model cheese and left to diffuse for 5 min throughout the surrounding medium, in the dark and in an air-conditioned room at 19°C. Solute diffusion into the model cheeses began as soon as the fluorescent solution was left in contact with the surface of the cheese (*t* = 0). The fluorescent solutes diffuse into the gel by a plane one-dimensional diffusion mechanism (Figure 1).

#### Time-lapse confocal laser scanning microscopy

Model cheese samples were imaged on an inverted NIKON Eclipse-TE2000-C1si microscope allowing confocal laser scanning microscopy (NIKON-France, Champigny sur Marne, France), with an oil-immersion 40 × objective at 512 × 512 pixel resolution. Ten minutes after the deposit of the fluorescent solution containing the diffusing solutes, the first step was to localize a fluorescently (SYTO9™) labeled colony, that had grown both in a focal plane at 10–15 μm depth from the coverslip and at a quite close distance from the surface of the gel, in order to visualize the diffusion front of fluorescence of the RITC-labeled solutes in a reasonable time-scale. SYTO9™ fluorescence was excited with the 488 nm laser and detected between 500 and 530 nm. The second step was to obtain the kinetic of the diffusion process of the RITC labeled solutes thanks to the acquisition of images of the fluorescent front of diffusion around and into the target bacterial colony every 5–15 min for at least 2 h. Fluorescently RITC-labeled dextrans and proteins were excited at 543 nm wavelength, and fluorescence emission was detected between 565 and 615 nm. All experiments were performed at 19°C using a temperature-regulated platform and air-conditioned room and were performed at least in triplicate.

#### Estimation of diffusion coefficients

Images obtained after a time series of acquisition were analyzed using ImageJ software. A line measuring 512 pixels long and 10 pixels wide was drawn through the axial diameter of the colony in order to quantify the profile of RITC fluorescence intensity (also named gray-level value) along the direction of the diffusion front and at each acquisition time. The resulting fluorescence intensity profiles vs. time were then exported into a single spreadsheet.

According to Crank (1975), in the case of a plane one-dimensional diffusion, induced by an instantaneous source and after a short time *t* of diffusion, the concentration gradient of the investigated solute is given by the following relationship:

(2) $$C(x,t)=\frac{M}{8\sqrt{\pi Dt}}exp\left(-\frac{x^{2}}{4Dt}\right)$$

where *C* is the concentration expressed as the amount of diffusing solute per unit area of surface, *x* is the perpendicular axis to the surface of the model cheese in μm (*x* = 0 corresponded to the surface), *t* is the time, *D* is the diffusion coefficient and *M* is the surface concentration of the diffusing solute, which corresponds to the total amount of the investigated fluorescently labeled solute in the gel related to the gel surface unit. This concentration *M* was considered as constant since it was in great excess compared to its concentration inside the cheese and as there was no reaction between the investigated component and the model cheese.

Equation (2) can be linearized:

(3) $$Ln\;\left(C(x)\right)=Ln\;\left(\frac{M}{8\sqrt{\pi Dt}}\right)-\frac{x^{2}}{4Dt}$$

The diffusion coefficient *D* was estimated from the slope of the straight line *Ln (C(x))* vs. *x*^2^, equal to −1/(4*Dt*). The slopes of the lines inside and outside the colonies were obtained by performing a linear regression with the best-fit linear trend function in Microsoft Excel using the least-squares method. Two effective diffusion coefficients *D*_out_ and *D*_in_ were quantified from the concentration profiles around the bacterial colony (*D*_out_) and inside the bacterial colony (*D*_in_). Both *D*_out_ and *D*_in_ were determined from the concentration profiles (gray values or fluorescence intensity profiles) obtained at a short time of diffusion, i.e., 30 min after the deposit, of the fluorescent solution at the surface of the model cheese.

### Statistical analysis

One-Way analysis of variance (ANOVA) and Tukey's paired comparison test were applied to the diffusion coefficient data in order to determine which mean values were significantly different from one another at the 95% confidence level using the R software package (version R i386 3.0.2).

## Results and discussion

### Surface of both lactoccoci strains are hydrophilic, but TIL1230 is more electronegative than LD61 at the pH of the model cheese

The first step of the strategy of this study was to determine the hydrophobic/hydrophilic character, Lewis acid-base interactions, and electrostatic cell surface properties. The MATS method and EM measurements gave us information on the potential ability of the two *L. lactis strains* to generate physicochemical interactions between both the cheese matrix and the diffusing solutes.

Results of the MATS method are reported in Table 2.

**Table 2:** Results of the microbial adhesion to solvents (MATS) method^*^.

***L. lactis* strain**	**% of adhesion to**	
	**Hexadecane**	**Chloroform**
LD61	8.6 ± 5.2 (*n* = 4)	11.3 ± 9.8 (*n* = 3)
TIL1230	12.8 ± 5.3 (*n* = 6)	13.7 ± 2.5 (*n* = 5)

* *The results are expressed as the mean ± one standard deviation of n independent measurements*.

The partitioning of cells between aqueous and hexadecane is a direct measurement of the cell surface hydrophobicity or hydrophilicity. The surface property of a cell can be considered as hydrophilic if its affinity for apolar hexadecane is below 40% (Giaouris et al., 2009). As shown in Table 2, the percentage of adherent cells to hexadecane was slightly higher (not significantly) for TIL1230 than for LD61, with values largely inferior to 20% for both *L. lactis* strains, demonstrating a clear hydrophilic character of their surface. The hydrophilic character of bacteria is largely due to the nature of the compounds present on the surface, useful for adhesion (Burgain et al., 2014).

The percentages of bacterial adhesion to the chloroform, an acidic solvent and electron acceptor, were not significantly different between the two *L. lactis* strains, with values also inferior to 20% (Table 2). These results are in agreement with values of adhesion to chloroform obtained on various *L. lactis* strains by Ly et al. (2006) and Giaouris et al. (2009).

**Figure 2:**
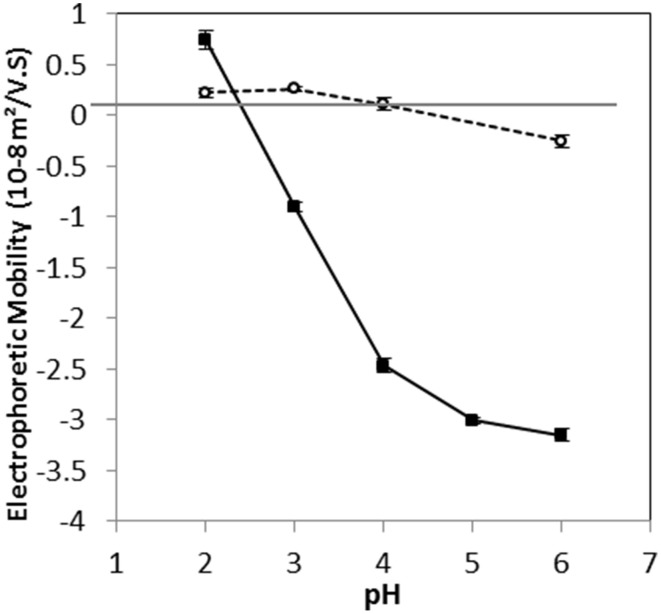
**Variation of electrophoretic mobility as a function of pH for *Lactococcus lactis* LD61 (circles) and TIL1230 (squares)**. Cells were harvested in the stationary growth phase. Measurements were made in 1 mM KNO_3_ solution on two independent sets of data; the bars represent the standard deviations of mean values.

The electrophoretic mobilities (EM) of the two *L. lactis* strains at different pH values indicated that the isoelectric points were around pH 2.5 and 4.5 for TIL1230 and LD61 strains, respectively (Figure 2). Between pH 2 and 6, the EM of *L. lactis* TIL1230 drastically decreased by about 4 × 10^−8^, whereas it decreased only 0.4 × 10^−8^ m^2^/V.s for LD61. *L. lactis* TIL1230 has a greater EM above pH 3 than LD61. Interestingly, LD61 strain presented EM very close to zero at all pH values tested, revealing very low electronegative cell surface in those conditions. In contrast, *L. lactis* TIL1230 was found to be highly negatively charged at pH between 4 and 6, as previously observed for most of *L. lactis* strains, with same order of magnitude for EM values, ranging from −2 to −5 × 10^−8^ m^2^/V.s (Ly et al., 2006; Habimana et al., 2007; Giaouris et al., 2009). Giaouris et al. (2009) reported for the first time that some lactic acid bacteria possess a very low surface electronegativity around neutral pHs, as observed here for *L. lactis* LD61. This diversity in the global charge of lactococcal cell surface may be linked to the variability of the molecules containing ionized groups in the cell envelope. Three types of ionized groups are considered to determine the surface electrical properties of *L. lactis*: phosphate groups present in teichoic and lipoteichoic acids, and carboxylate and protonated amino groups of proteins (Boonaert and Rouxhet, 2000). It has been previously shown that the expression of the major cell wall-anchored protease was responsible for altering *L. lactis* surface physicochemical properties, shifting the cell envelope from a hydrophilic surface to an extremely hydrophobic one, going along with an increase of negative charges at the cell surface (Habimana et al., 2007). In the present study, the two strains of *L. lactis* LD61 and TIL1230 were thus supposed to present greater differences in their cell surface properties as TIL1230 does not possess the cell wall protease and LD61 does. Obviously, the global property of the cell surface is multi-causal and then difficult to predict.

Bacterial cell wall properties were shown to affect diffusion particles of nanoparticles inside biofilm matrices of *L. lactis* (Habimana et al., 2011). They measured that the diffusion of 50-nm radius particles of anionic carboxylate-modified fluorescent polystyrene beads was more hindered in biofilm matrix of *L. lactis* which possessed the anchored protease. Based on these results, the present study aimed at comparing the diffusion of solutes of different charge, flexibility, and hydrophobicity, inside immobilized colonies (in cheese) of two different *L. lactis* strains presenting (LD61) or not (TIL1230) the anchored protease in their cell walls.

### Diffusion coefficients of dextrans inside the colonies depend on the solute size but not on the lactococci strains

**Figure 3:**
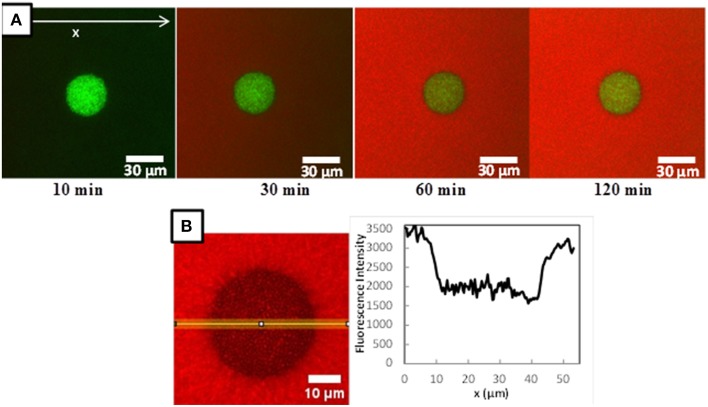
**(A)** Example of a time-lapse microscopic observations of a *Lactococcus lactis* LD61 colony immobilized in a model cheese after 15 h growth at a temperature of 30°C during diffusion of a RITC-labeled dextran (here RITC-Dextran 70 kDa). *L. lactis* cells are colored in green, RITC-dextran in red. **(B)** Focus on the microscopic observation of the fluorescence intensity of RITC-dextran inside and around a *L. lactis* LD61 colony immobilized in a model cheese at *t* = 120 min of diffusion and the corresponding fluorescence intensity profile along a 10 pixels wide line vs. position x. Black zones in the colony corresponds to the *L. lactis* cells.

**Figure 4:**
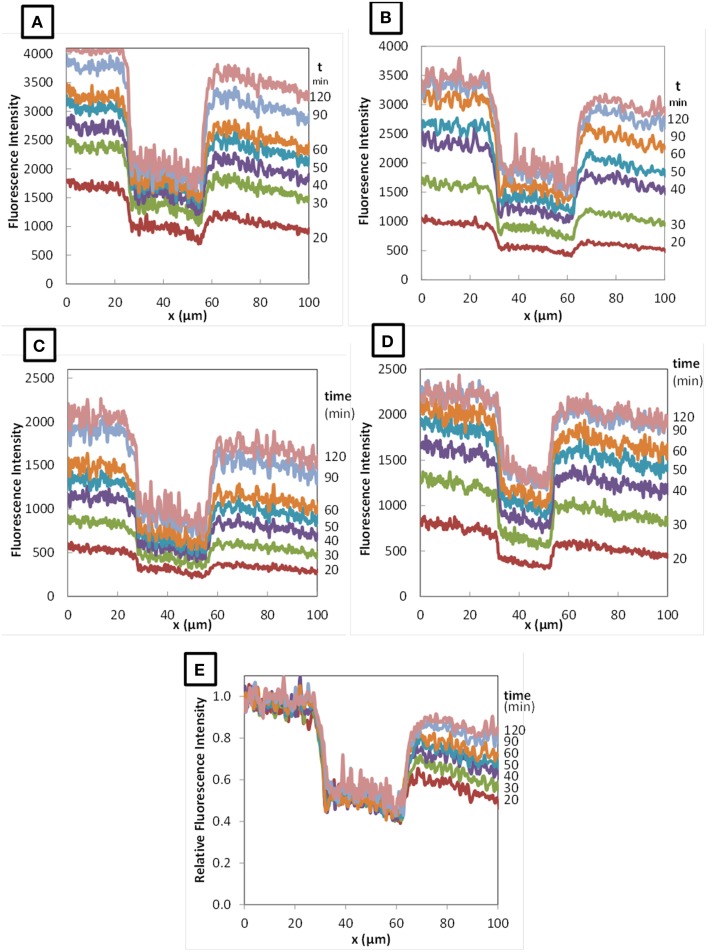
**Profiles of fluorescence intensity during diffusion of RITC-dextran 10 kDa (A), 70 kDa (B), and 155 kDa (C) in colonies of *Lactococcus lactis* LD61 and of RITC-dextran 70 kDa in TIL1230 (D) as function of diffusion time (from 20 to 120 min)**. **(E)** Typical relative fluorescence intensity profile obtained with RITC-dextran 70 kDa in LD61.

Figure 3A shows typical confocal microscopic observations of *L. lactis* colonies, immobilized in the model cheese, and visualized at different times after the deposit of the 70 kDa fluorescently-labeled dextran solution. The images obtained with *L. lactis* TIL1230 and the two other fluorescently-labeled dextrans (10 and 155 kDa) were similar and are thus not shown here. Both lactococci strains grew in this model cheese as perfect spheres with diameters around 30–40 μm, as previously reported in Jeanson et al. (2011) and Floury et al. (2013). Figure 3A also clearly shows the progressive increase of the red fluorescence both along the x-axis and through time, proving that the fluorescent solute progressively moved inward toward a Lactococcus colony because of the concentration gradient between the surface and the interior of the model cheese. After 2 h of diffusion (Figure 3B), the red color was uniform in all the directions around the colony, meaning that the concentration of the fluorescently-labeled solute had reached a plateau. The diffusing process ended because there was no more concentration gradient in this area. The simple observation of these time series of images also suggests that the hypothesis of unidirectional diffusion of solute is valid. Therefore, image analysis of the intensity profiles of red fluorescence along this x-axis allowed to directly quantifying the diffusive penetration of the solutes as a function of time (Figure 3B). Typical fluorescence intensity profiles were obtained (Figures 4A–C) at different times (between 20 and 150 min). Only an example of the fluorescence intensity profiles obtained with TIL1230 strain was shown on Figure 4D because the profiles were very similar to those obtained with LD61.

From all these fluorescence intensity data as a function of the position and the time of diffusion, we calculated the corresponding relative fluorescence intensity by dividing the fluorescence intensity at each position x by the average fluorescence intensity obtained on the region upstream the colony. An example is given on Figure 4E for the diffusion of the 70 kDa dextran in a colony of *L. lactis* LD61. The relative fluorescence intensity profiles obtained with the other dextrans and with the other strain were very similar (data not shown). The purpose of this graphical representation of the results was to observe the evolution of the ratio of the fluorescence intensity in the colony vs. outside the colony as a function of time.

As shown on Figure 4E, whatever the time of diffusion considered, the fluorescence intensity inside the colony of both *L. lactis* strains drastically dropped compared to the fluorescence intensity in the surrounding cheese matrix, with a quite constant ratio around 0.4–0.6 depending on the x-axis position inside the colony. We previously showed that even the fluorescently-labeled solutes diffuse inside a bacterial colony (Floury et al., 2013), but do not penetrate into the bacterial cells. Furthermore, dextrans are not metabolized by lactococci cells. Thus, the only way to explain this drop of relative fluorescence intensity inside the colony is because on the line of 10-pixel wide (Figure 3B), the volume filled with the fluorescent solution is lower inside the colony than outside the colony due to the presence of the bacterial cells (corresponding to black zones with no fluorescence intensity). So even if the experimental fluorescence intensity is different, the real concentration of the fluorescent solute is effectively the same both inside and around the colony. The calculated average value represented by the relative fluorescence intensity profiles was quite stable along the x-axis inside the colonies for the fluorescently-labeled dextran solutes (Figure 3B), and this was also true whatever the time of diffusion considered (Figure 4E).

**Figure 5:**
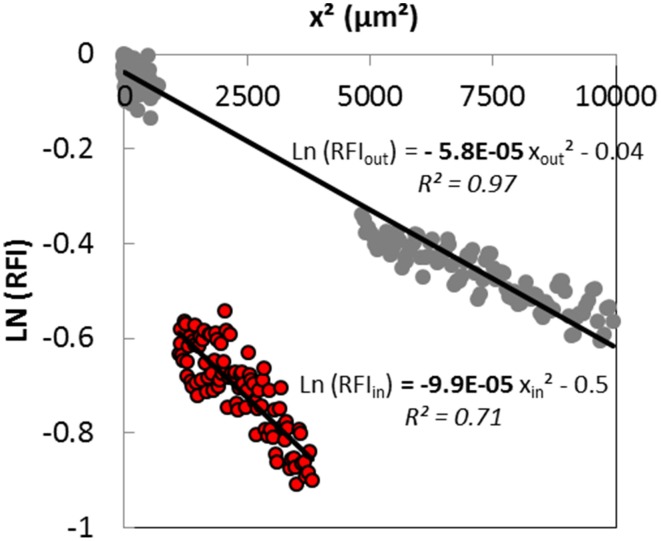
**Typical linearized curve obtained 30 min after the deposit of the fluorescent solution containing a 70 kDa dextran at the surface of the model cheese inoculated with *Lactococcus lactis* LD61**. Lines correspond to the linear fit of experimental data both outside (gray circles) and inside the colony (red circles).

The concentration of the diffusing solute cannot be calculated from experimental data. The fluorescent intensity is, however, proportional to the diffusing solute concentration. Then, assuming a one-dimensional Fickian diffusion induced by an instantaneous source, the effective diffusion coefficients both inside the colony and in the surrounding matrix could be estimated from the slope of the linearization of the experimental fluorescence intensity profiles obtained a short time after the beginning of the diffusive process, with $D=-\frac{1}{4.t.slope}$ from Equation (3). A typical linearized curve and the corresponding fitted equations obtained both outside and inside the colony are shown on Figure 5. Regression coefficients of the linear models were generally higher than 0.9 in the surrounding matrix, and slightly lower inside the colony with values around 0.7 because of a lower signal to noise ratio inside colonies. The slope of the line was clearly higher inside than outside the colony (Figure 5), suggesting that the diffusion of solutes was slower inside the colony. The diffusion of the fluorescent solute was probably more hindered inside the colony, most likely because of the high volume filled with the bacterial cells, than in the protein-network of the surrounding matrix.

**Figure 6:**
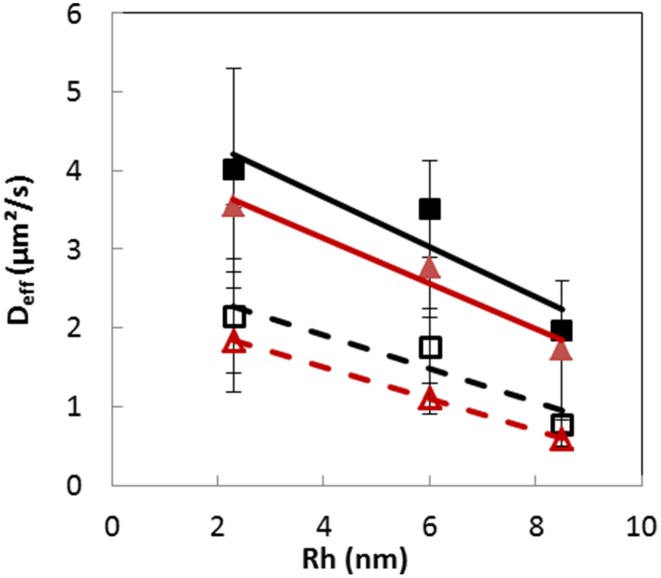
**Plots of the mean effective diffusion coefficients (D_eff_) of dextrans 10, 70, and 155 kDa vs. their respective hydrodynamic radius, estimated from experimental data obtained after 30 min, both inside (open symbols) and outside (plain symbols) colonies of *Lactoccus lactis* LD61 (squares) and TIL1230 (triangles) in a model cheese**. Lines, linear regression.

The mean effective diffusion coefficients (*D***_eff_**) of the different RITC-dextrans were obtained using this modeling approach, both inside and outside the colonies of the two bacterial strains (Figure 6). *D*_eff_ were significantly (*p* < 0.05) lower inside the colonies than in the surrounding matrix. When *D*_eff_ were plotted against the hydrodynamic radius, linear relationships (*R*^2^ > 0.8) were obtained over the molecular weight range of 10–155 kDa, both inside and outside colonies. The statistical analysis (ANOVA) performed on the estimated values revealed that the values of *D*_eff_ were not significantly different (*p* < 0.001) between the two bacterial strains, meaning that dextran solutes diffused inside both Lactocci colonies with a similar diffusion coefficient, which depended mainly on size of the solute. The absence of significant difference between both *L. lactis* strains was expected because dextrans are known to be hydrophilic macromolecules and the surface properties of the two different cells were shown to be also both very hydrophilic.

In the present study, we went further than the previous study (Floury et al., 2013) by visualizing the kinetic of the diffusion process inside the colonies. Moreover, we were able to estimate diffusion coefficients of the dextran solutes inside the microbial colonies from image analysis of the data. As shown in Table 3, whatever their size between 10 and 155 kDa and the *Lactoccocus* strain, the effective diffusion coefficients of dextrans obtained inside the colonies were around twice lower than their respective diffusion coefficients in the surrounding cheese matrix around the colonies, and up to 50 times smaller than those in water *D*_w_ (calculated from the Stokes-Einstein equation). Silva et al. (2013) also found that effective diffusion coefficient values of fluorescein isothiocyanate (FITC)-dextrans (from 4 to 2000 kDa) in the same model cheese, but not inoculated, were smaller than those in water due to the hindrance of the protein network. However, their values of effective diffusion coefficients were between 4 and 9 times larger than in the present study, depending of the size of the dextran.

**Table 3:** Calculated ratios of effective diffusion coefficients of dextrans obtained inside and outside *L. lactis* colonies (*D*_in_/*D*_out_), of diffusion coefficients of dextrans in water^*^ vs. inside colonies (*D*_w_/*D*_in_), and corresponding tortuosity indices, calculated as square root of *D*_w_/*D*_in_.

**Dextran MW (kDa)**	***D*_in_**/*D*_out_		*D*_w_^*^/*D*_in_		***Tortuosity index***	
	***L. lactis* strain**					
	**LD61**	**TIL1230**	**LD61**	**TIL1230**	**LD61**	**TIL1230**
10	0.54	0.52	43	50	6.6	7.1
70	0.50	0.40	20	32	4.5	5.7
155	0.40	0.34	32	43	5.7	6.5

* *Dw values at 20°C calculated by using the Stokes-Einstein relationship*.

The variability observed has two major causes. The first source of variability is the experimental approaches used to estimate diffusion coefficients (Floury et al., 2010). Effective diffusion coefficients estimated thanks to the concentration profiles method in the present method are then difficult to compared to the so called “self-diffusion” coefficients obtained using the FRAP technique in Silva et al. (2013).

The second source of variability is the model cheese. Indeed, even if the model cheese had the same initial composition in both studies, the metabolism of the lactococci inoculated in the present study induced a strong decrease of cheese pH from 6.6 to 5.1–5.3, depending on the *L. lactis* strain, which probably modified to some extent the microstructure of the protein network and then the diffusion behavior of the solutes in both cheeses.

In agreement with our results, Guiot et al. (2002) and Thurnheer et al. (2003) observed that the diffusion coefficients of fluorescently-labeled dextrans from 3 to 70 kDa also decreased linearly with hydrodynamic radius in different mono- and poly-species biofilms, and were up to 150 times smaller than those in bulk water. However, the direct comparison of their results with our study is difficult because the microbial cell distribution is rather different in a biofilm and in a food matrix such as cheese. To our knowledge, only two studies are realistically comparable to our system (Rani et al., 2005 and Takenaka et al., 2009). They focused the analysis of the solute diffusion exclusively within identified clusters of microbial cells inside different model oral biofilms. The diffusive penetration of two tracer molecules (rhodamine B and fluorescein, MW~400 Da, chosen as model of antibiotic for their similar size), into staphylococcal cell clusters was directly visualized by confocal scanning laser microscopy (Rani et al., 2005). The effective diffusion coefficients of the two fluorescent tracers were around 10 times lower than the corresponding solute diffusion coefficient in water. The difference with the present study could be due to a denser population of cells in our colonies. The diffusive penetration of fluorescently-labeled dextrans of various molecular weights (from 3 to 70 kDa) was visualized into three different species of cell clusters formed by oral bacteria grown in a flow cell (Takenaka et al., 2009). Like in the present study, the effective diffusion coefficient of dextrans strongly decreased with their molecular weights. However, their order of magnitude was different from our results, with effective diffusion coefficients only twice smaller than those in water. For Thurnheer et al. (2003), analysis of diffusion phenomena within biofilms suggested tortuosity as the most probable factor responsible for retarded diffusion compared to water. They defined a tortuosity index, as the square root of *D*_w_/*D*_in_, representing an indicator of solute diffusion through interstitial space between bacterial cells. A molecule going through a highly convoluted three-dimensional route in a matrix will be delayed in comparison with free diffusion in water. As shown in Table 3, the tortuosity indexes estimated from our experimental data were of the same order of magnitude regardless of the *L. lactis* strain and the size of the diffusing dextran solutes. In our conditions, the extracellular space between the lactococci cells within the colony is filled with an aqueous phase composed of water, lactose, and minerals (Floury et al., 2013), thus explaining why the tortuosity index did not depend on the size of the dextrans.

In conclusion about the diffusion of dextrans within bacterial colony, we confirmed that macromolecules as large as dextrans of 155 kDa diffused into lactococci colonies in a model cheese (Floury et al., 2013). We demonstrated that their diffusion was similar for two different strains of lactoccoci whatever the size of dextrans up to 155 kDa.

### Milk proteins, such as BSA, lactoferrin and α_S1_-casein, do not diffuse inside neither LD61 nor TIL1230 lactococci colonies

**Figure 7:**
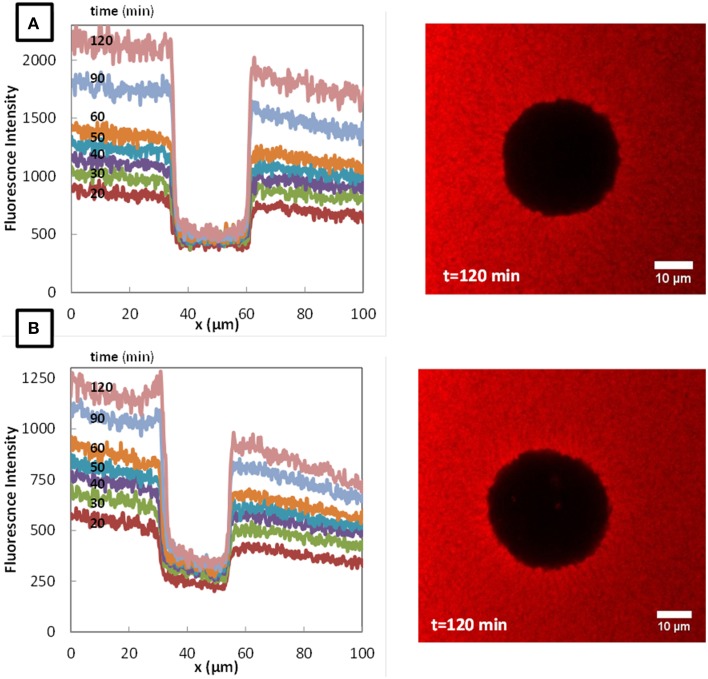
**Fluorescence profiles of fluorescently-labeled Bovine Serum Albumin (BSA), in colonies of *Lactococcus lactis* LD61 (A) and TIL 1230 (B) at different times from 20 to 120 min after the deposit at the surface of a model cheese and the corresponding microscopic observations of the colony after 120 min of diffusion**.

**Figure 8:**
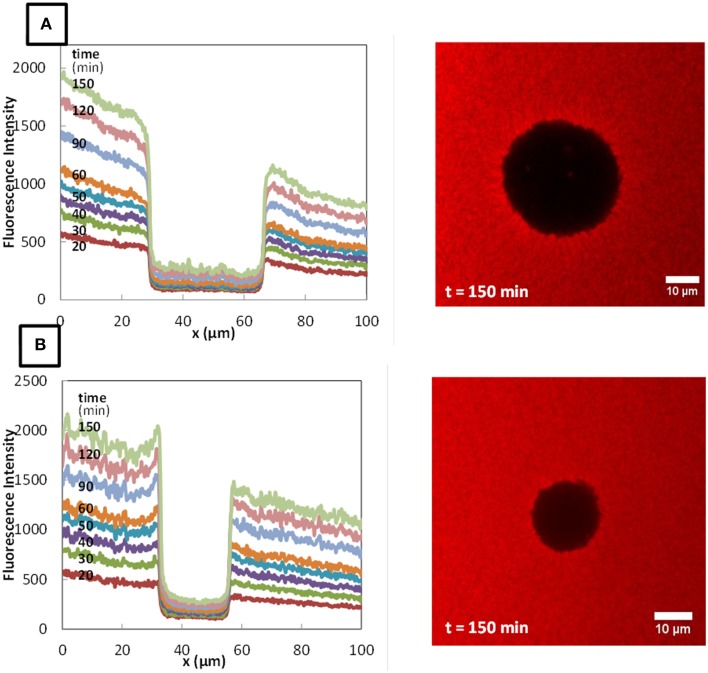
**Fluorescence profiles of RITC-LF in *Lactococcus lactis* LD61 (A) and TIL1230 (B) from 20 to 150 min and corresponding microscopic observations of the colony after 150 min of diffusion**.

**Figure 9:**
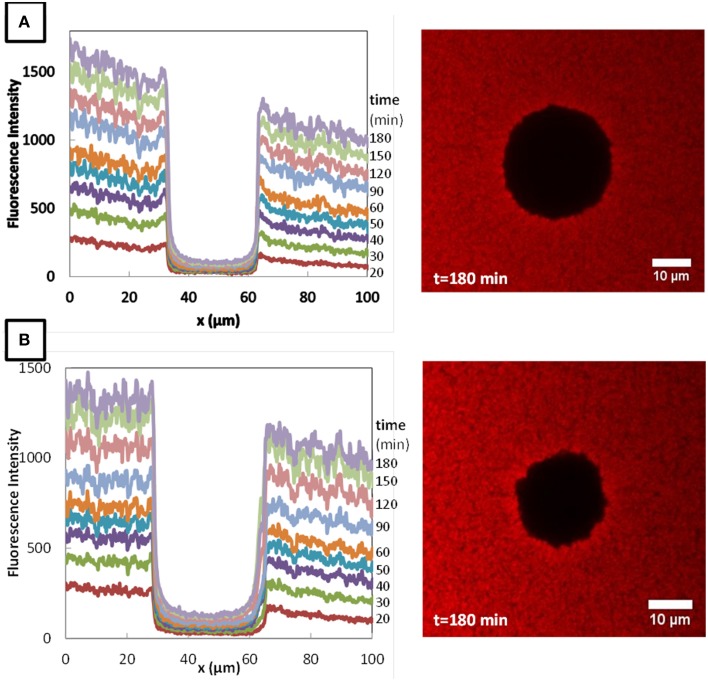
**Fluorescence profiles of RITC-α_S1_-casein in *Lactococcus lactis* LD61 (A) and TIL1230 (B) from 20 to 180 min and corresponding microscopic observations of the colony after 180 min of diffusion**.

The typical fluorescence intensity profiles for three fluorescently-labeled proteins, and typical images of the corresponding colonies at the end of the experiments are shown on Figures 7–9. Whereas, the fluorescence intensity of the three proteins outside the colonies increased with time, the fluorescence intensity measured inside the colonies was very low all along the duration of the experiments. The increase of the fluorescence intensity throughout time outside the colonies shows that the three labeled-proteins effectively diffused in the surrounding cheese matrix. Whereas, the fluorescence outside the colonies was finally intense, the observation of the micrographs clearly confirmed the intensity profiles by the absence of fluorescence inside the colonies. This means that, surprisingly, none of the three proteins could diffuse inside the bacterial colonies, although their hydrodynamic radii were much smaller than the radius of the largest dextran (Table 1). Concerning the diffusion of proteins in cell clusters, to our knowledge the only published study is from Takenaka et al. (2009). Contrary to our results, they clearly visualized by time-lapse confocal microscopic observations that fluorescently-labeled proteins, even the largest like ConA (MW 104 kDa) and IgG (MW 150 kDa), diffused inside microbial cell clusters that were approximately a few hundred micrometers in diameter and reached the center of these cell clusters in less than 3 min.

These results highlighted that the size of the diffusing solute was not the sole factor conditioning its ability to enter inside colonies of *L. lactis* immobilized in cheese. Other physicochemical factors such as flexibility, charge and/or hydrophobicity of the solute can also be of involved, by generating bacteria–solute interactions of different nature, especially for diffusing solutes such as milk proteins. Moreover, the impact of these factors can also depend on the surface properties of the strain (Habimana et al., 2011).

When diffusing through a non-inoculated model cheese, the rigid, and globally negatively charged BSA protein was hindered more than dextrans with a similar hydrodynamic radius, because of the existence of solute-matrix interactions (Silva et al., 2013). In the same way, deBeer et al. (1997) and Takenaka et al. (2009) reported that diffusion coefficients of solutes in different kinds of cell clusters were conditioned on the network structure in the interstitial space, but also mainly depended on the size and the charge of the diffusing solute. Unlike dextrans that are flexible, neutral, and hydrophilic polymers, proteins possess different shapes, hydrophobicity, and charges (Table 1). Moreover, even if the surface of the cells of the two *L. lactis* strains were shown to be both hydrophilic, *L. lactis* TIL1230 was more electronegative than LD61 at the pH of the model cheeses. Therefore, we could have expected the surface properties of bacterial cells to influence the ability of solutes to diffuse or not inside the colony by generating either repulsive or attractive interactions like electrostatic forces, depending on the charge of the solute (Burgain et al., 2014). The pH of the model cheeses were around 5.1 and 5.3 for LD61 and TIL1230, respectively. Therefore, according to the isoelectric point of the proteins (Table 1), the net charges of BSA and α−_S1_ casein were slightly negative, whereas LF was globally positively charged in both cheese matrices. Electrostatic repulsions could have then occurred between the outer bacterial cells of the colony and the negatively charged solutes, preventing their diffusion inside colony. However, it is largely known that cheese is a medium presenting a high ionic strength (around 100 mM). In that case, the Debye length is very small (around 2 nm) and then the energy barrier due to repulsive forces is very low, meaning that the electrostatic contribution is strongly suppressed (Burgain et al., 2011). Interactions of electrostatic nature were not explaining the non-ability of the three milk proteins to penetrate inside *L. lactis* colonies immobilized in cheese. Other kind of repulsive forces such as hydrophobic interactions can be involved, especially between the hydrophobic BSA and LF proteins and the hydrophilic surfaces of the cells of both *L. lactis* strains. However, for the amphiphilic α−_S1_ casein, there was no reason for repulsions of hydrophobic origin with the bacterial surfaces.

We were finally not able to explain why α−_S1_ casein proteins could not diffuse inside the lactococci colonies. However, it is well-known that milk caseins, especially the α−_S1_ casein, are hydrolyzed by the cell wall proteases of *L. lactis* in cheese and/or other proteases present in the cheese. We can thus hypothesize that some of the generated peptides can diffuse inside colonies, and are further metabolized into smaller peptides and amino acids by all the cells located inside the colonies, as strongly suggested by the results obtained in the same model cheese by Le Boucher et al. (personal communication).

## Conclusion

Effective diffusion coefficients of dextran macromolecules were quantified for the first time inside colonies of two different *L. lactis* strains immobilized in a model cheese. We clearly showed that the diffusion behavior of macromolecules through bacterial colonies immobilized in a model cheese not only depends on the size of the diffusing solutes, but also and mainly on their physicochemical properties. Whereas, a flexible and neutral hydrophilic polymer such as a dextran can diffuse inside colonies whatever its size, none of the three proteins investigated in this study could penetrate inside lactococci colonies. These original results remain unexplained because both the surface of the two bacterial strains and the three diffusing proteins presented various physicochemical properties, from rigid to flexible shapes, and from negatively to neutral and positively charged. Our results finally show that the choice of the fluorescently-labeled molecule as a model of diffusing solute is crucial.

### Conflict of interest statement

The authors declare that the research was conducted in the absence of any commercial or financial relationships that could be construed as a potential conflict of interest.
